# Superior anti-neoplastic activities of triacontanol-PEG conjugate: synthesis, characterization and biological evaluations

**DOI:** 10.1080/10717544.2018.1477864

**Published:** 2018-07-19

**Authors:** Yimeng Zhou, Ning Li, Zhixia Qiu, Xiaoyu Lu, Min Fang, Xijing Chen, Lili Ren, Guangji Wang, Pingkai Ouyang

**Affiliations:** a China Pharmaceutical University, Nanjing, China;; b Nanjing Tech University, Nanjing, China

**Keywords:** PEGylated triacontanol, triacontanol, micelle, drug delivery, anti-neoplastic

## Abstract

Triacontanol (TA, C_30_H_62_O), abundantly present in plant cuticle waxes and bee waxes, has been found to display promising anti-neoplastic potentials. As a long chain fatty alcohol, TA possesses limited aqueous solubility, which hinders its medicinal application. To overcome its solubility barrier, a polymer prodrug was synthesized through attaching TA to poly ethylene glycol (PEG), using succinic acid as a linker with bifunctional amide and ester bonds. Anti-neoplastic effects of PEG-TA were assessed in LoVo and MCF7 cells, anti-proliferative and apoptosis-inducing activities were subsequently confirmed in mouse xenograft model. Encouragingly, PEG-TA possessed selective anti-cancer ability. It did not exhibit significant cytotoxicity on normal cells. Mechanistic examination revealed inhibition of NF-κB nuclear translocation, suppression on matrix degradation enzyme and down-regulation of angiogenic signaling might contribute to its anti-malignant effects. Pharmacokinetics clearly indicated PEGylated TA (named as mPEG2K-SA-TA) substantially enhanced TA delivery with increased plasma exposure (19,791 vs. 336.25 ng·mL^−1^·h^−1^_,_*p* < .001), mean residence time (8.46 vs. 2.95 h, *p* < .001) and elimination half-life (7.78 vs. 2.57 h, *p* < .001) compared to those of original TA. Moreover, mPEG2K-SA-TA appeared to be safe in preliminary toxicological assessment. PEGylated TA also emerged as a functional carrier to deliver hydrophobic chemotherapeutic agents, since it readily self-assembled to micelles in aqueous solution with a low critical micelle concentration (CMC, 19.1 µg·mL^−1^). Conclusively, PEG-TA conjugate displayed superior anti-neoplastic activities and low toxicity, as well as facilitated the delivery of other hydrophobic agents, which appeared to be an innovative strategy for cancer therapy.

## Introduction

Triacontanol (TA, C_30_H_62_O), a long and normal chain fatty alcohol, is traditionally applied as a growth stimulant and pesticide in the field of agriculture (Naeem et al., 2012). Also, it is widely used as nutritional supplements (Fan et al., 2006; Dullens et al., 2008; Kim et al., 2017). Importantly, TA exhibits no toxic effects on humans or animals, no contaminative risk to environment since it mostly exists in waxy plants and insects (Singh et al., 2006). Recently, it has been reported that TA could inhibit the proliferation and metastasis of cancer cells, via inhibiting cyclooxygenase 2 (COX-2) and vascular endothelial growth factor (VEGF) (Zhang et al., 2016). Furthermore, it could promote spleen lymphocyte proliferation, then improve immune system via activating natural killer cells and facilitate macrophage phagocytosis. When applied in combinational therapy, TA significantly confront against leukopenia induced by cyclophosphamide. Despite its potential in cancer treatment, the wide application of TA is impeded by its limited water-insolubility due to its highly lipophilicity and large structure, which result in poor absorption and low bioavailability, thus diminishing its health promoting effects (Naeem et al., 2012; Gong et al., 2017). Moreover, TA is rapidly cleared with short resident time evidenced in previous investigations (Wang et al., 2015). Therefore, numerous efforts have been exerted to improve its solubility, enhance its bioavailability and alleviate its pharmacokinetics by various pharmaceutical formulation technologies, aiming to encapsulate TA and deliver it into target organ by polymers. However, there have been as yet no reports on pharmacokinetics improvements by attaching TA to hydrophile polymers to overcome its poor aqueous solubility.

Poly ethylene glycol (PEG) is such kind of polymer, with nontoxic, nonimmunogenic, nonantigenic properties, and has been approved by US Food and Drug Administration (FDA) for drug delivery (Veronese & Pasut, 2005; Bailon & Won, 2009; Li et al., 2013). To date, PEGylated proteins, peptide, antibody fragments, and oligonucleotides have been approved to execute clinical trials (Pasut & Veronese, 2009; Li et al., 2013; Hamley, 2014; Stefan et al., 2014; Senevirathne et al., 2016). PEGylated prodrugs were mainly designed to improve the pharmaceutical properties of their parent forms (solubility, stability or permeability) with simple structural modification (mainly conjugation) (Mahato et al., 2011; Abet et al., 2017). TA is a high aliphatic alcohol and extremely hard to disperse in water phase (2 × 10^−16 ^M or 9 × 10^−14 ^g·L^−1^), thus it obtains low tissue permeability (Naeem et al., 2012). The hydroxyl group in its structure is the only potential binding site for carboxyl group to form ester prodrug, which is supposed to be chemically or enzymatically transformed to TA after administration based on the prodrug strategy. Therefore, a dedicated study was executed to attach TA to PEG.

At present study, a series of PEGylated TA prodrugs (mPEG with different molecular weight, including mPEG1K, 2K, 5K, 10K) were initially synthesized by a simple solution phase reaction. Based on in-lab *in vitro* cytotoxicity assay and preliminary pharmacokinetics comparison, the optimized PEGylated TA, mPEG2K-SA-TA was chosen for subsequent *in vivo* and *in vitro* anti-tumor efficacy assessment, uptake mechanism study as well as anti-tumor mechanism investigation. Afterwards, a comprehensive pharmacokinetic study was carried out to further support the *in vivo* and *in vitro* pharmacological assay of mPEG2K-SA-TA. Also, hemolytic test and acute toxicity assay were carried out to evaluate the safety of mPEG2K-SA-TA. Finally, as a conjugate polymer, mPEG2K-SA-TA exhibited amphipathic property and probably functioned as a drug vehicle. Hereby, a series of experiments were carried out to investigate the feasibility of the optimized PEGylated conjugate as a drug carrier.

The present study innovatively attempted to chemically modify TA through attaching PEG to overcome its solubility barrier based on prodrug strategy, to achieve the superior anti-neoplastic activities of TA. Meanwhile, the pharmacokinetics, pharmacodynamics, and safety evaluation of the newly prepared triacontanol derivative was performed to demonstrate the advantages of the compound. As an amphipathic PEG conjugate, we further anticipate the novel PEGylated TA would provide an ideal solution to carry hydrophobic cancer chemotherapeutic agents.

## Materials and methods

### Materials

TA (purity >95%) was kindly offered by kindly offered by Clinical Pharmacokinetics Research Laboratory, China Pharmaceutical University (NanjingNa, China). Monomethoxy Poly (ethylene glycol) amine 2 K (mPEG2K-NH_2_) used to graft with succinic acid (one end formed amide and the other exposed carboxyl**)** was purchased from Ponsure Company (Shanghai, China) with poly dispersity around 1.05, the purity (>95%) was testified by ^1^H-NMR. 1-Ethyl-3-(3-dimethylaminopropyl) carbodiimide (EDCI), 4-dimethylaminopyridine (DMAP), N, N-Diisopropylethylamine (DIPEA) were purchased from Sigma-Aldrich (St. Louis, MO, USA). Succinic acid (SA) was purchased from J&K Chemical Ltd. (Shanghai, China). mPEG2K-SA-TA (NMR purity, 95.0%) was prepared and purified by ToYongBio Ltd. (Shanghai, China). The HPLC grade solvents used (methanol, acetonitrile and heptane) were purchased from Tedia (Fairfield, OH, USA). The derivatization reagent, N, O-Bis (trimethylsilyl) trifluoroacetamide (BSTFA) was purchased from Aladdin (Shanghai, China). Distilled water was prepared using a Milli-Q water purification system. Ammonium acetate was supplied by Sinopharm Chemical Reagent Co., Ltd (Shanghai, China). All other chemicals and agents were of analytical grade from Nanjing Chemical Reagent No. 1 Factory (Nanjing, China).

The primary antibody of VEGF (sc-7269, 21 kDa) and Histone H1 (sc-8030, 34 kDa) were purchased from Santa Cruz Biotechnology (Santa Cruz, CA, USA), NF-κB (#4764S, 65 kDa) and β-actin (# 4967S, 45 kDa) were supplied by Cell Signaling Technology (Beverly, MA, USA), MMP9 (ab38898, 92 kDa) was kindly offered by Abcam plc (Cambridge, MA, USA). The goat anti-rabbit IgG-HRP (sc-2004, used for the WB analysis of MMP, NF-κB, β-actin) and goat anti-mouse IgG-HRP (sc-2005, used for WB analysis of VEGF and Histone H1) were purchased from Santa Cruz Biotechnology (Santa Cruz, CA, USA).

### Synthesis of PEGylated TA

**Figure 1.**
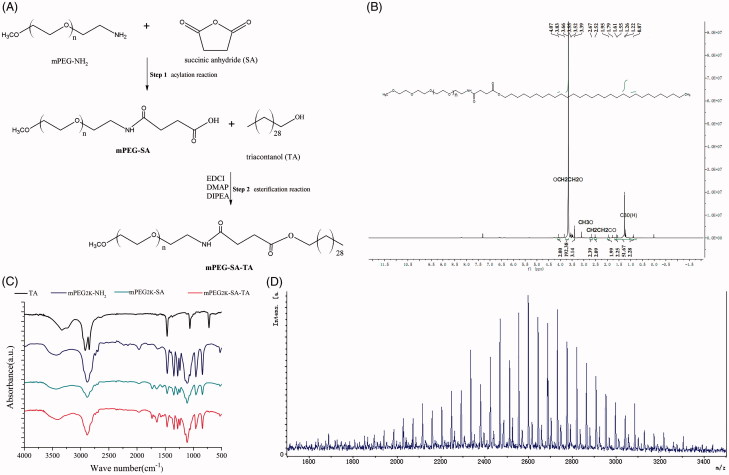
Synthesized process of PEGylated TA and structural characterization of PEGylated TA (mPEG2K-SA-TA) by ^1^H-NMR spectrum, FT-IR spectrum, MALDI-TOF spectrum.

The below scheme in Figure 1(A) summarized synthesized diagram of mPEG2K-SA-TA, consisted of the sequential acylation reaction and esterification reaction. The detailed preparation was conducted as following.

### First-step: synthesis of mPEG2K-SA

mPEG2K-NH_2_ (0.5 g, 1 eq) and an aliquot of 20 mL anhydrous acetonitrile was added to a round-bottomed flask. The mixture was cooled to −5 °C with magnetic stirring under nitrogen. Afterwards, succinic anhydride (SA, 0.55 g, 20 eq) was added in batches. When the temperature of the mixture spontaneously reached room temperature, it was stirred overnight. On the next day, the mixture was concentrated up to dryness, and then 50 mL of dichloromethane and 30 mL of deionized water was added. The reaction mixture was then adjusted to pH 7.0 with 2M NaOH and continuously stirred for 1 h to break the excess amount of SA. And then the system was adjusted to pH 3.0 with 6M hydrochloric acid. After separation, the organic phase was collected. The aqueous phase was re-extracted with 50 mL of dichloromethane and the collected organic phase were combined. Dried with anhydrous sodium sulfate, the crude products were loaded on silica gel column (100–200 mesh, developing solvent dichloromethane:methanol 15:1) and to give 0.4 g of mPEG2K-SA (Yield, 76.20%) after elution.

### Second step: synthesis of mPEG2K-SA-TA

mPEG-SA (0.5 g, 1 eq), TA (0.5 g, 5 eq), EDCI (2 eq), DIPEA (3 eq), DMAP (0.05 g) were dissolved in 20 mL dichloromethane and stirred overnight at room temperature. EDCI was used as dehydrating agent to activate carboxyl and promote the formation ester bond. DMAP was the catalyst of esterification. As an alkaline reagent, DIPEA was used as nucleophilic reagent and acid binding agent. On the next day, another aliquot of 50 mL dichloromethane was added into the flask, followed by washing with water (twice). Afterwards, the organic phase was dried by anhydrous sodium sulfate, loaded and purified on silica gel column (100–200 mesh, dichloromethane:methanol 15:1 as developing reagent) to harvest the off-white final compound 0.51 g (yield, 82.6%).

### Chemical characterized analysis of synthesizedmPEG2K-SA-TA

^1^H-NMR (Nuclear Magnetic Resonance Spectroscopy) studies were carried out to confirm the PEGylation. The compounds were dissolved in deuterated chloroform and analyzed by Oxford AS400 NMR spectrometer (Oxford, UK) operating at 400 MHz and spectral processing with chemical shifts reported in ppm. The chemical structures of target conjugate were characterized by IR-Tracer 100 FT-IR analysis (Fourier transform infrared spectroscopy) (Shimadzu, Japan). Samples were directly mixed with potassium bromide (KBr) to form homogeneous KBr pellet and scanned for absorbance ranging from 4000 to 500 wavenumbers (cm^−1^). Whether TA was successfully grafted with mPEG was confirmed by matrix-assisted laser desorption/ionization time of fight mass spectrometry (MALDI-TOF; Bruker Daltonics, USA). The samples were dissolved in deionized water and used 2,5-dihydroxybenzoic acid (DHB) as assisted matrix.

### In vitro cytotoxicity study of mPEG2K-SA-TA

The 3-(4,5-dimethylthiazol-2-yl)-2,5-diphenyltetrazolium bromide (MTT) assay was introduced to evaluate the anti-tumor potency of synthesized PEG-TA conjugate. Human breast cancer cell lines (MCF-7) and human colon carcinoma cells (LoVo) cell lines were utilized to evaluate the *in vitro* cytotoxicity, and L02 cell lines (normal human liver cell) were used to assess the *in vitro* safety of target polymers. Cells were cultured in standard DMEM (MCF-7 and L-02) or DMEM-F12 (LoVo) supplemented with 10% (v/v) FBS and 1% (v/v) penicillin–streptomycin solution. All cell lines were grown in the incubator (5% CO_2_) at 37 °C. Concretely, the cells were seeded in a 96-well plate and cultured for 24 h in 200 μL medium/cell with a density of 5 × 10^3^ cells/well at 37 °C. Serial levels of PEGylated TA solutions were separately added into each cell (ranging from 0.11 to 2283 μM). After 48 h co-incubation, the incubation mixtures were withdrawn and washed by PBS for three times. An aliquot of 200 μL MTT solutions (0.5 mg/mL) was added to each well and incubated for another 4 h at 37 °C. Afterwards, the MTT solutions were abandoned and another 150 μL DMSO was added to dissolve formazan. The absorbance of the wells was measured by the microplate reader (Eon, BioteK, USA) at 490 nm. The cell viability was defined as the absorbance ratio between the wells incubated with PEGylated TA and control wells with culture medium. The inhibitory potency was evaluated by 50% inhibitory concentration (IC_50_) and estimated by GraphPad Prism 5 (GraphPad Software, La Jolla, CA, USA).

### In vitro cell apoptosis assay

The LoVo cells were cultured as the procedure in MTT assay. The cells were seeded in six-well plates at a density of 5 × 10^5^/mL. When the cells were grown to 90% confluence (about overnight), different levels of mPEG2K-SA-TA (200 and 500 μg/mL) were added and incubated for 4, 12, 24, 48 h (buffer only, no drugs as control group). At the end of culture, a volume of 5 μL of Annexin V-EGFP and 5 μL of Propidium Iodide were gently mixed with 100 μL of cell suspension and incubated for 15 min at room temperature in the dark. Finally, an aliquot of 400 μL of binding buffer was added and apoptosis was detected by flow cytometry (FACS Calibur, Becton-Dickinson, USA).

### In vivo anti-tumor evaluation of mPEG2K-SA-TA in xenograft tumor models

The tumor inhibition efficacy was evaluated in female BALB/c nude mice bearing LoVo cells. Female BALB/c nude mice (18-20g) were kindly supplied by KeyGEN BioTECH (Nanjing, Jiangsu, China). The nude mice xenograft tumor models were constructed by subcutaneously inoculated with LoVo cells (1 × 10^6^ cell/mouse, 0.1 mL/mouse) at right axilla. The mice were randomly divided into four groups (10 mice in each group). The mice in negative group were treated with an equal aliquot of physiological saline (0.1 mL/10g body weight, every two days), while mice in other groups received isometric volume of PEGylated TA solution (400 or 500 mg/kg body weight, 0.1 mL/10 g body weight, daily i.v administration) or cisplatin injection (3 mg/kg body weight, 0.1 mL/10 g body weight, every two days via i.v administration). Throughout the entire experimental period, the tumor sizes (diameters) and the body weights were carefully monitored and recorded every two days. Finally, the nude mice were sacrificed and these vital tissues, including tumor, heart, liver, lung, kidney and spleen were immediately harvested. The weights and diameters of tumors were recorded. The volumes of tumors were calculated using the formulation: *V*= (length × width^2^)/2, length and width are the longest and the shortest diameter of the tumor, respectively. The relative tumor volume (RTV) was calculated as RTV = *V*_t_/*V*_0_, where *V*_0_ represents the initial tumor volume (designated as day 0, prior to drug treatment) and *V*_t_ denotes the tumor volume of specified day (after drug administration). The tumor growth inhibitory rate (IR) was introduced as IR = (tumor weight_neg_−tumor weight_treatment_)/tumor weight_neg_, in which tumor weight_neg_ is the average tumor weight in mice treated with physiological saline and tumor weight_treatment_ represents the average tumor weight in mice treated with synthesized polymer or cisplatin.

In order to better support the anti-tumor evaluation and following histological assay, the harvested tissues were prepared as abovementioned method to monitor the amount of TA by GC–MS/MS in these tissues, including heart, liver, spleen, lung, kidney and tumor.

### Histology and immunohistochemistry

The histology indicated by hematoxylin & eosin (H&E) staining was used to assay the potential tissue toxicity of mPEG2K-SA-TA. The harvested tissues were separately fixed in 4% paraformaldehyde for 48 h, followed by embedded in paraffin after dehydration and sliced into pieces (thickness, 0.4 μm). And then the slices were stained with H&E and observed by light microscope (NIKON Eclipse ci, Japan) and further photographed (NIKON digital sight DS-FI2, Japan).

The immunohistochemical analysis was carried out to investigate the anti-cancer activity of mPEG2K-SA-TA, including the straining of terminal deoxynucleotide transferase-mediated dUTP nick-end labeling (TUNEL), Ki-67 and CD31. Specifically, TUNEL was to evaluate the tumor cell apoptosis, while Ki-67 and CD31 were used to assess the tumor cell proliferation, using the labeled streptavidin–biotin method.

### Effects of mPEG2K-SA-TA on the translocation of NF-κB to nucleus

As reported, octacosanol could effectively inhibit the proliferation of endothelial cells and ascites cancer cells, suppress angiogenesis and secretion of cancerous ascites, which were largely pertinent to the inhibition of NF-κB translocation. TA, as a homolog of octacosanol, may also exert tumor killing through a similar mechanism. Therefore, it was necessary to investigate the anti-tumor activity of PEGylated TA and its possible mechanism.

In consideration of the role of NF-κB in facilitating tumor metastasis and promoting tumor angiogenesis(Li et al., 2012), matrix metalloproteinase-9 (MMP-9) and vascular endothelial growth factor (VEGF) are also investigated as typical down-stream of NF-κB regulation, both are regarded as potential effect target of TA (Wang et al., 2006; Pan et al., 2012). NF-κB, VEGF, and MMPs are critically involved in the processes of tumor cell invasion and metastasis (Raina et al., 2013). As similar procedure in MTT assay, the LoVo cell line was used to examine the potential role of mPEG2K-SA-TA in the inhibition of translocation of the transcription factor (NF-κB) to the nucleus, to exhibit its anti-tumor activity *in vitro* and *in vivo*.

### Western blot analysis

The procedure of western blot analysis was depicted in supporting information for western blot analysis. The nucleoprotein was extracted for the translocation analysis of NF-κB, using Histone H1 as reference protein, while other proteins were analyzed using β-actin as reference protein.

### Real-time qPCR analysis

The procedure of western blot analysis was depicted in supporting information for Real-time qPCR analysis. The primer sequences of both MMP9 and VEGF were shown in Table S1.

### Rationalize the mPEG2K-SA-TA design in view of pharmacokinetics comparison

Sprague–Dawley (SD) rats (male and female, 300 ± 30 g) were purchased from SIPPR/BK Experimental Animal Co., Ltd (Shanghai, China). All animals were kept in a humanized environment, with the temperature maintained at 20 ± 2 °C and relative humidity at 50 ± 10%. All the animal procedures were performed following the approved protocol. SD rats were randomly divided into different groups (*n* = 4 in each group). Rats from different groups were intravenously (i.v.) administrated with TA solution, PEGylated TA solution mPEG2K-SA-TA, respectively. At the designated time points, the blood samples were collected into polyethylene tubes containing EDTA at 0, 0.083, 0.167, 0.5, 0.75, 1, 2, 4, 8, 12, 24, 36 and 48 h and then centrifuged at 12,000 rpm for 5 min to obtain plasma. The plasma samples were stored at −80 °C till analysis. The harvested plasma samples were prepared by sample direct saponification or extraction and analyzed by GC–MS/MS (Wang et al., 2015). The determination method was initially validated and satisfied with the criteria of the linearity, precision and accuracy, extraction recovery, matrix effect and stability under various conditions.

The pharmacokinetic parameters were calculated by a noncompartmental analysis using WinNonlin (Version 6.4; Pharsight, Mountain View, CA, USA). The maximal plasma concentration (*C*_max_) and time reaching the summit concentration (*T*_max_) were directly observed from the original data. The systemic exposure to TA (area under the plasma concentration versus time profile from the dosing time to the terminal sampling time, AUC_0−t_) was calculated by trapezoidal rule. Along with the *in vitro* MTT assay, the pharmacokinetic process of TA was compared among different synthesized polymer prodrugs to rationalize the PEGylated prodrug design and support the *in vivo* anti-cancer efficacy evaluation.

### Safety assay of mPEG2K-SA-TA

#### Hemolytic effect of mPEG2K-SA-TA

Red blood cells (RBCs) freshly collected from rats were used for hemolytic analysis. The immediately harvested RBCs were washed with cold physiological saline and diluted to 2% w/v. The serial amounts of mPEG2K-SA-TA (1, 2, 5 and 10 mg/mL) were added to the diluted RBCs suspension. Meanwhile, the RBCs treated with saline solution or distilled water was served as negative or positive control. After incubation at 37 °C for 4 h, the mixtures were centrifuged at 1500 rpm for 10 min to collect the supernatants, which were pipetted to 96-well plate to monitor the absorbance of released hemoglobin (OD value) at 545 nm. The hemolytic effect was evaluated by hemoglobin release and calculated as the following equation.

$$\text{ Hemoglobin release(\%)}=\frac{{\text{OD}}_{\text{sample}}-{\text{OD}}_{\text{control}}}{{\text{OD}}_{\text{positive}}-{\text{OD}}_{\text{control}}}$$

#### In vivo acute toxicity of mPEG2K-SA-TA

BALB/c mice were supplied by SIPPR/BK Experimental Animal Co., Ltd (Shanghai, China). The mice were equally divided into two groups (*n* = 6, male and female), one group was treated with physiological saline as control group, while the other received i.v. administration of mPEG2K-SA-TA. After a single dose administration, the mice were further kept for 14 successive days. At the end of experiment, the mice were sacrificed to collect the vital organs, heart, liver, spleen, lung and kidney. Meanwhile, the blood or serum were collected to immediate blood test or serum biochemical index analysis with HITACHI 7170 Automated analyzer (Hitachi, Japan), including WBC, white blood cell; RBC, red blood cell; HGB, hemoglobin; PLT, platelet; ALT, alanine aminotransferase; AST, aspartate aminotransferase; BUN, Urea nitrogen; CREA, Creatinine.

#### Preparation and characterization of mPEG2K-SA-TA micelle

Due to the amphiphilicity, mPEG2K-SA-TA was well dissolved in deionized water and characterized as micelles. The particle size distribution of the formed micelles was determined by Quasi-elastic laser light scattering via a zeta plus dynamic light scattering (DLS) detector (Brookhaven Instruments Co. USA) at 25 °C.

The critical micelle concentration (CMC) of synthesized polymers was determined by pyrene as a fluorescence probe. The stock solution of pyrene was prepared in acetone at 6 × 10^−6 ^M. After the evaporation of acetone under nitrogen stream, an aliquot of separate PEGylated TA solution with serial concentrations was added to produce the final concertation at 0.05, 0.1, 0.2, 0.5, 1, 2, 5, 10, 20, 50, 100, 200, 500, 1000, 2000, 5000, 10,000 and 20,000 μg/mL. After ultrasonic treatment for 30 min, the solutions were kept in water bath at 70 °C for 1 h to reach equilibrium. The fluorescence spectra were monitored by a luminescence spectrometer (RF-6000, Shimadzu, Japan). The fluorescence intensity ratio of I_338_/I_333_ was analyzed versus the PEGylated TA concentration, and the abrupt point of fluorescence intensity ratio of I_338_/I_333_ was recognized as the CMC value.

The morphologies of PEGylated TA micelle were investigated using transmission electron microscope (TEM) (HT7700 Exalens, Hitachi, Japan). The samples were added to copper grids, and then stained with 2% (w/v) phosphotungstic acid. After natural drying, the samples were observed by TEM.

#### Cellular uptake of mPEG2K-SA-TA micelle

As a prodrug, the *in vitro* release profile of TA from mPEG2K-SA-TA was an important criterion to subsequently evaluate its anti-tumor potency. The synthesized PEGylated TA was prepared in phosphate buffer solution (PBS, 0.01M) under different pH condition (pH =5.0, 7.4) and incubated at 37 °C with the final concentration at 1 mg/ml. At designated time points, an aliquot of 100 μL incubation mixture was transferred and analyzed by GC–MS/MS after sample preparation.

The cellular uptake assay was carried out in LoVo cells with Coumarin-6 as a hydrophobic fluorescence probe. The LoVo cells were seeded on the sterile coverslips at the bottom of six-well plates with a density at 5 × 10^5^ cells/well, with 1 mL complete DMEM/F12 medium. After the cells reached 80% confluence (incubation 24 h), the culture medium was replaced by blank mPEG2K-SA-TA solution, Coumarin-6 micelles with Coumarin-6 concentration at 0.25 mg/mL, free Coumarin-6 solution and incubated for 1 h and 4 h at 37 °C. Subsequently, the incubation media were abandoned and carefully washed with PBS for three times. Afterwards, the cells were fixed with 4% paraformaldehyde and the nucleus was stained with DAPI (2.5 μg/mL) for 25 min. Finally, the coverslips were taken out and covered on glass slides to study the intracellular uptake by confocal laser scanning microscopy (CLSM, LSM 700, Carl Zeiss, Germany).

Using the similar procedure, the cells treated with Coumarin-6 solution and mPEG2K-SA-TA micelle loading Coumarin-6 were kept at 37 °C for 4 h, afterwards, the incubations were digested by 0.25% trypsin (free EDTA) and suspended by PBS. And then, the intracellular uptake of Coumarin-6 was monitored by flow cytometer (FACS Calibur; Becton-Dickinson, USA).

#### Endocytosis mechanism of mPEG2K-SA-TA

The LoVo cell line was used to investigate the uptake mechanism of PEGylated TA micelle. LoVo cells were seeded with a density at 5 × 10^5^ cells/well in a 24-well plate. After incubation at 37 °C for 24 h, the cells were ready for uptake mechanism assay with various inhibitors, including sodium azide (NaN_3_, 1.5 mg/mL), chlorpromazine (5 µg/mL), colchicine (8 µg/mL) and nystatin (20 µg/mL). Meanwhile, another plate was incubated at 4 °C for 30 min, then about 1 mL of PEGylated TA micelle was added into the plate to investigate whether the uptake was energy-dependent. After incubation for 4 h, the media were removed and washed with PBS for three times, and then an aliquot of water was added into each well and followed three circles freeze-thaw and further tore cells apart by ultrasonic processor. One part was used to protein concentration by BCA assay (kits from Nanjing Jiancheng Bioengineering Institute, Nanjing, China), while the other part was used to measure the intracellular amounts of TA by GC–MS/MS.

#### Application of mPEG2K-SA-TA micelle to deliver docetaxel

Since mPEG2K-SA-TA could easily self-assemble to micelle in aqueous solution, it was also believed to encapsulate hydrophobic drugs. Docetaxel (DTX) was used as the representative drug. By simple thin-film hydration method, DTX and mPEG2K-SA-TA formed the thin film after the evaporation process. Afterwards, the film was dissolved in PBS to self-assemble to micelle. After 0.45 μm filtration, the particle size, zeta potential and morphology of micelle loading DTX was analyzed as abovementioned. The entrapment efficiency and drug loading capacity was evaluated according to our published method (Yang et al., 2017). The micelle loading DTX was administrated to rats by i.v. bolus administration. The pharmacokinetic process of DTX were compared between DTX solution and DTX micelle (1 mg/kg). The blood samples were collected at 0.08, 0.17, 0.50, 0.75, 1.00, 2.00, 4.00, 8.00, 12.00, 24.00, 36.00 and 48.00 h and then centrifuged at 12,000 rpm for 5 min to obtain the plasma. The plasma samples were stored at −80 °C until LC–MS/MS analysis. The pharmacokinetic parameters of each formulation were calculated using a noncompartmental model by WinNonlin as abovementioned process.

#### Statistical analysis

Results were depicted as mean ± SD (standard deviation). The statistical analysis was run by one-way ANOVA test. When *p* < .05, *p* < .01, *p* < .001, statistical difference was taken into consideration.

## Results and discussion

### Rationalization and structural characterization of mPEG2K-SA-TA

Linear PEG is one of the most widely used polymers for its superior water solubility, the repeating ethylene oxide subunits in the backbone could generate H-bond with water in aquatic environment (Kadajji & Betageri, 2011). In commercial market, several PEG derivatives were available. One simple way was directly binding TA with carboxylated PEG to generate PEG-TA ester conjugate using methylcarboxyl as linker, in which one terminal hydroxyl group of PEG was oxidized to a carboxylic acid (Wichitnithad et al., 2011). The activated PEG derivative, methoxypolyethylene glycol amine (mPEG-NH_2_) is a better choice to be readily linked with small molecule drugs with only one binding site, since one end is capped with methoxy group, the other is potentially linked with an active amido. Moreover, the spacer or linker is also necessary and functions as an important factor in constructing a prodrug conjugate. Several linker groups could be chosen to conjugate with parent drugs, which usually contain bifunctional coupling sites, such as amino acids, anhydride. As a traditional linker, succinic acid (SA) is widely applied in the area of protein modifications for its no-toxic, inert specialty(Shen et al., 2013). Two carboxy groups make it prone to link with amino or hydroxyl to form amide or ester linkage.

Traditionally, one carboxyl in SA is coupled with the terminal hydroxyl in PEG, while the other one is linked with target molecular to generate a prodrug with double ester bonds (Chu et al., 2016). However, due to stronger p–π conjugative effect in amide, it is widely accepted that amide has a relatively higher enzymatic stability than ester bond. Rather than the double-ester bonds, we preferred to the amid-ester structure, aimed to improve its chemical stability. Hence, amide bond was initially synthesized ahead of ester bond. In reality, if esterification reaction was firstly conducted and followed by acylation reaction, the ester bond would be easily broken during aminolysis.

As depicted in the synthesized scheme (Figure 1(A)), mPEG2K-NH_2_ along with succinic anhydride were used as the starting materials to conjugate with TA by simple two-steps solution phase reaction, including acylation and esterification reaction in sequence. As shown in ^1^H-NMR spectrum of PEGylated TA, the signals at 3.66 and 3.39 ppm are attributed to the methylene protons and terminal methoxyl protons of mPEG, while 1.26 and 0.87 ppm belong to the methylene protons and terminal methoxyl protons of TA, as shown in Figure 1(B). In FT-IR spectra, as depicted in Figure 1(C), the characteristic peaks appear at 1650 and 1735 cm^−1^, attributed to carbonyl group in amide bond and terminal carboxy group (—COO—, mPEG2K-SA,) or esteratic linkage (—CO—NH—, mPEG2K-SA-TA), while the representative peaks of carbonyl group are not observed in the spectrum of PEG amine or TA. In the MADLDI-TOF analysis, the molecular weight (MW) of mPEG2K-NH_2_, mPEG2K-SA-TA determined by MALDI-TOF was 2480, which was close to the theoretical MW, there showed also repetitive units, ethyoxyl (MW, 44) in TOF spectrum (Figure 1(D)). All these characteristic peaks in IR spectrum, ^1^H-NMR spectrum as well as MALDI-TOF indicated the successful synthesis of mPEG-SA and mPEG-SA-TA, with relatively high production ratio (above 80%, yield).

### In vitro cytotoxicity assay of mPEG2K-SA-TA

**Figure 2.**
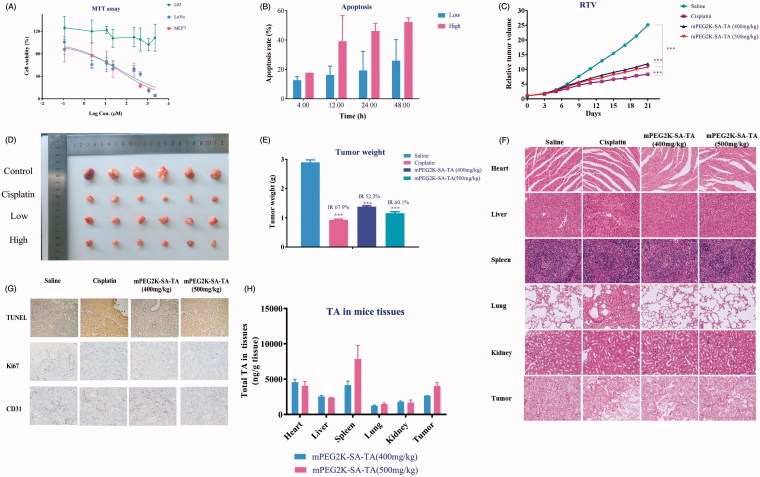
Cytotoxicity assay ofmPEG2K-SA-TA on L02, MCF7 and LoVo (A); Apoptotic rates (%) induced by mPEG2K-SA-TA in medium for 4, 12, 24 and 48 h determined by FCM (B); Changes of relative tumor volume after treatment with saline, cisplatin, mPEG2K-SA-TA (400 or 500 mg/kg, *n* = 10) (C); Representative photos of tumor tissues at the end of experiments (D); The weights of excised tumors from different groups of nude mice bearing LoVo tumor (E); H&E staining of vital organs after treatment with saline, cisplatin, 400 and 500 mg/kg mPEG2K-SA-TA (F); Representative TUNEL, CD31 and Ki67 immunohistochemical images of tumors from different groups (G); Tissue distributions of TA into heart, liver, spleen, lung and kidney after treatment with saline, cisplatin, 400 and 500 mg/kg mPEG2K-SA-TA (H) *p < .05, **p < .01, ***p < .001 vs. control group.

The anti-cancer effect evaluations were carried out in MCF-7, LoVo and L02 cell lines, respectively. The PEGylated TA (mPEG2K-SA-TA) showed no obvious cytotoxicity (ranging from 0.11 to 2283 μM) on normal L02 cell lines but significant cytotoxic effect on MCF-7 and LoVo cell lines, with the estimated IC_50_ at 45.5 and 56.69 μM, respectively. When the amount of PEGylated TA reached 1141 μM (500 μg/mL, TA equivalent), the mortality was up to above 80%. The cytotoxicity was probably pertinent to TA, released from PEGylated TA. Along with the increment of TA amounts, the cellular viability was gradually decreased, as shown in Figure 2(A). While for the direct TA testing, we failed to get the IC_50_, due to the poor solubility (data not shown) and could not reach the equivalent amount of PEGylated TA.

The cytotoxicity assay revealed that the PEGylated TA had no toxic effect on the normal cells (L02) but selectively inhibit the growth of MCF7 and LoVo cells, the two-representative human tumor cell lines. Therefore, the PEGylated TA polymers exhibited superior anti-neoplastic activities on tumor cells.

### *In vitro* cell apoptosis assay of mPEG2K-SA-TA

After double staining with Annexin V-PI, the apoptosis state was analyzed by flow cytometry. The lower left quadrant represents viable cells (designated as Q4), the lower right quadrant denotes early apoptotic cells (named as Q2), and the right upper quadrant represents late apoptotic cells (called as Q3). The total apoptosis rate was calculated as the sum of Q2 and Q3. As summarized in Figure 2(B), mPEG2K-SA-TA induced early apoptosis and late apoptosis of LoVo cells in a time-dependent manner. Up to 48h, the apoptosis rate reached 52.34% when PEG2K-SA-TA was at 500 μg/mL.

### *In vivo* anti-tumor efficacy of mPEG2K-SA-TA

Based on the MTT evaluation and apoptosis assay, the *in vivo* anti-tumor efficacy assay was further investigated in nude mice bearing LoVo tumor model with mPEG2K-SA-TA. As shown in Figure 2(C), the tumor sizes were increased as time went onwards, but the tumor growth was significantly inhibited by daily administrated mPEG2K-SA-TA, compared to the mice treated with physiological saline **(**Photos of tumors in Figure 2(D)). Unlike the dramatic body weight decrease in cisplatin group (positive control), mPEG2K-SA-TA did not obviously downregulate the body weight, in other words, the polymer was well tolerated after consecutive i.v. injections for 21 days (Figure S1). At the end of drug intervention, the tumor weight in PEGylated TA groups (1.38 ± 0.90 g for low dose and 1.16 ± 0.15 g for high dose, *p* < .05) were significantly lighter than that in saline group (2.90 ± 0.20 g, *p* < .001). By daily 400 and 500 mg/kg PEGylated TA treatment, the tumor growth inhibition rate (IR) was 52.3% and 60.1%, respectively (Figure 2(E)). These results suggested that mPEG2K-SA-TA was efficient in suppressing tumor growth with lower systemic toxicity.

### Apoptosis and anti-proliferation of mPEG2K-SA-TA

Further H&E staining and immunohistochemical analysis were conducted to support its *in vivo* safety and potency, as shown in Figure 2(F and G). The TUNEL staining assay was used to evaluate the apoptosis of LoVo cells by analysis of DNA fragmentation in nucleus of tumor cells (Yang et al., 2017). The apoptotic tumor cells stained brown were observed under observation. More apoptosis was observed in polymer groups compared to saline group as shown in Figure 2(G). Afterwards, Ki-67 and CD31 were introduced to evaluate the proliferation of LoVo cells. (Ki-67 indicates the proportion of cells in proliferative cycle and the faster of tumor proliferation; CD31 primarily demonstrates the presence of endothelial cells and microvascular density of tumors) (Croix et al., 2000; Jalava et al., 2006). After long-term treatment by mPEG2K-SA-TA, CD31 was decreased in group treated with PEGylated polymers (Figure 2(G)), the expression of Ki-67 was also down-regulated in some extent (Figure 2(G)). The apoptosis and proliferation evaluation indicated that mPEG2K-SA-TA showed the anti-cancer ability via the upregulated of apoptosis and downregulated proliferation of LoVo cells, which was in accordance with the *in vitro* cell apoptosis assay.

Compared to the saline group, mPEG2K-SA-TA (400 and 500 mg/kg) showed no obvious histopathologic changes in heart, liver, kidney, spleen and lung despite considerable amounts of TA were found in heart, liver, spleen, lung, kidney (Figure 2(F)). TA was also detectable in tumor, which further incurred tumor cells necrosis (Figure 2(H)). These findings indicated that mPEG2K-SA-TA boasted superior anti-tumor potency but with no obvious toxicity to vital organs.

Moreover, the tissue exposure to TA in spleen ranked the largest, reaching 4171 ng/g tissue. And the spleen serves as the largest immune organ and participates in various immune responses. TA has also been reported to significantly promote the proliferation of mouse spleen lymphocytes and participate in the body’s immune regulation. Therefore, the largest distribution to spleen was of great significance in improving the immune function and exerting anti-tumor activity.

### Regulation of NF-κB translocation to nucleus by mPEG2K-SA-TA

**Figure 3.**
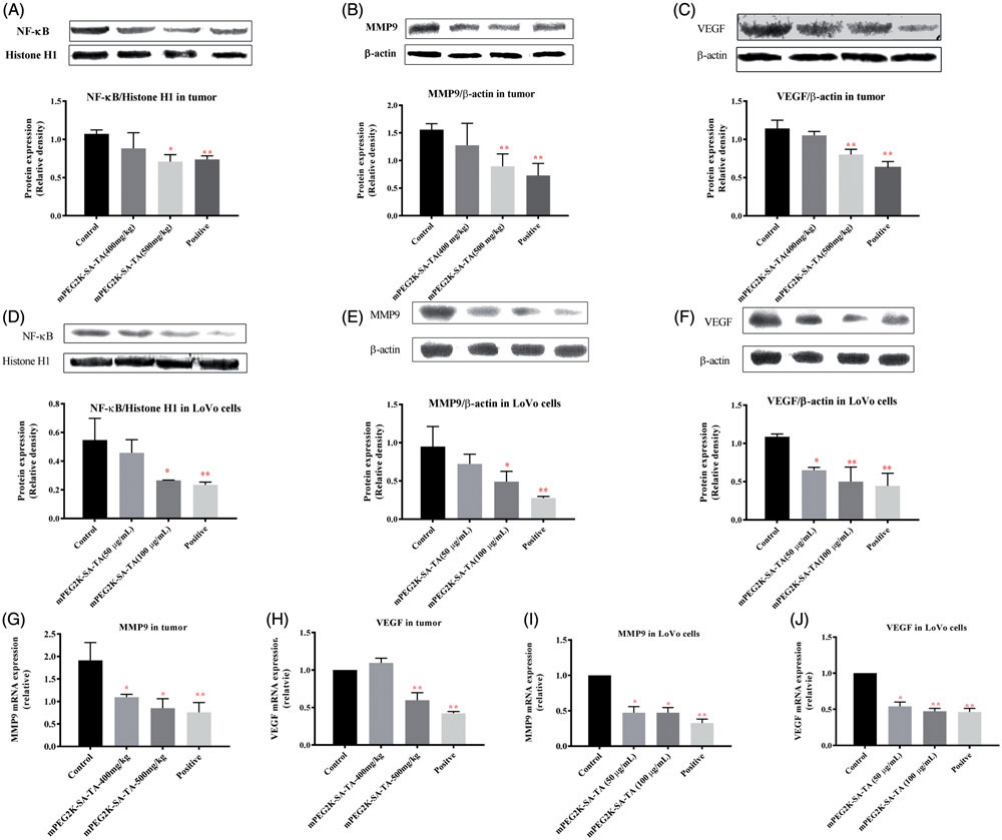
Western blot and qPCR analysis of tumors and cells. NF-κB translocation to nucleus was suppressed by mPEG-2K-SA-TA treatment in tissues from nude mice bearing LoVo cells (A) and *in vitro* LoVo cells (D), the nuclear protein was analyzed by Western blot using Histone H1 as reference protein; The expression of MMP9 was obviously inhibited by mPEG-2K-SA-TA in tissues from nude mice (B) and *in vitro* LoVo cells (E); The protein expression of VEGF was also decreased by mPEG-2K-SA-TA in tissues from nude mice bearing LoVo cells LoVo cells (C) and in vitro LoVo cells (F); Both MMP9 and VEGF were measured by Western blot using β-actin as reference protein. mRNA expressions of MMP9 and VEGF in tumor tissues (G, H) from nude mice bearing LoVo cells and *in vitro* LoVo cells (I, J) determined by Q-PCR. *p < .05, **p < .01, vs. control group.

Recently, it has been increasingly reported that octacosanol exerts its anti-cancer efficacy by down-regulating vascular endothelial growth factor (VEGF) through the inhibition of translocation of NF-κB to nucleus (Thippeswamy et al., 2008). Sharing similar chemical structure and physicochemical property, TA is also believed to possess comparable anti-tumor potency (Fan et al., 2006), probably involving NF-κB translocation and MMPs inhibition. It is widely recognized that NF-κB plays a vital role in promoting tumor metastasis (DiDonato et al., 2012), which further promotes the expression of MMP-9, one gene greatly related to tumor metastasis (Wang et al., 2006). Also, NF-κB in tumor cells strongly induces the expression of VEGF and facilitates angiogenesis (Shibata et al., 2002). In the present *in vitro* LoVo cells culture, the translocation of NF-κB to nucleus was significantly inhibited by PEGylated TA, as depicted in the Western blot analysis (Figure 3(A)). As the target gene of NF-κB, the expression of MMP9 was also down-regulated, at the protein, evidenced by the western blot analysis (Figure 3(B)). In tumor cells without treatment, the expression of VEGF was abundantly expressed (Figure 3(C)), which was also inhibited by mPEG2K-SA-TA (*p* < .05, PEGylated TA vs. saline).

We also compared the protein expression of NF-κB, MMP9 and VEGF in tumor tissues of nude mice bearing LoVo cells. In accordance with the *in vitro* results, PEGylated TA was able to inhibit the translocation of NF-κB to cellular nucleus (*p* < .05, 400 and 500 mg/kg groups vs. saline group). Likewise, the downregulated expressions of MMP and VEGF were closely pertinent to the reduced translocation of NF-κB produced by mPEG2K-SA-TA, as depicted in Figure 3(D–F).

At the mRNA level, the expression of MMP9 and VEGF were significantly down-regulated in tumor tissues isolated from nude mice bearing LoVo cells (Figure 3(G,H)). Likewise, both the gene expressions were also decreased in LoVo cells treated with mPEG2K-SA-TA, as depicted in Figure 3(I,J).

In order to further support our findings, the tumor tissues from tumor-bearing nude mice were subjected to immunohistochemical analysis of MMP9 and VEGF. The immunohistochemical analysis was performed on the nucleus of brown–yellow granules as positive staining. Light staining or no staining was judged as a negative staining result. The positive rate in the saline group was high, but the expression of MMP9 and VEGF could be significantly reduced by mPEG2K-SA-TA (as shown in Figure S2).

Comprehensive consideration of both *in vivo* and *in vitro* findings, it was convincing that mPEG2K-SA-TA promoted apoptosis and suppressed proliferation probably involving the decreased translocation of NF-κB to nucleus, further reduced expressions of MMP9 and VEGF at both mRNA and protein expression levels.

### Rationalized PEGylated TA design in perspective of pharmacokinetic comparisons

**Figure 4.**
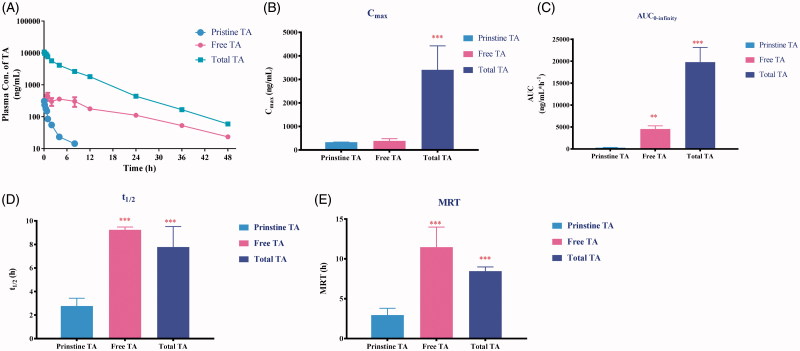
Plasma concentration–time profiles (A) and pharmacokinetic parameters (*C*_max_, *t*_1/2_, AUC_0−t_ and MRT, correspondently depicted as B, C, D and E, respectively) of pristine TA after i.v administration of TA solution (3 mg/kg), free TA and total TA after direct extraction or saponification following i.v. administration of mPEG2K-SA-TA (TA equivalent, 3 mg/kg), **p < .01, ***p < .001.

After i.v. administration to rats, the amounts of free TA and total TA were monitored and compared with pristine TA. After drug administration of mPEG2K-SA-TA, TA was immediately detected in rat plasma at the first sampling time point (0.083 h) with the *C*_max_ at 387.5 ng/mL, as depicted in Figure 4. The systemic exposure to released TA was significantly enhanced to 4552 ng·mL^−1^·h^−1^, far overweighed that from direct administrated equivalent TA solution (AUC_0−t_, 336.25 ng·mL^−1^·h^−1^, data from samples after saponification). Also, the released TA exhibited slow elimination rate (*t*_1/2_, 9.24 h). It also exhibited prolonged within-body resident time indicated by the MRT_last_ (11.5 h). While the full scheme of TA-time profile in rats treated with pristine TA was failed to get by direct extraction method, since quite a small portion of TA appeared in plasma as free form and only detectable in the front time points, while it was mainly conjugated with fatty acid as ester form (Paik et al., 2008; Haim et al., 2009).

We also compared the pharmacokinetic process of total TA (both free and conjugated forms). The system exposure to released TA from PEGylated TA (19791 ng·mL^−1^·h^−1^) severely surpassed that from direct administrated equivalent TA solution (*p* < .001, AUC_0−t_, 336.25 ng·mL^−1^·h^−1^). Also, the half-life time (*t*_1/2_, 7.78 h) was prolonged compared with original TA (*p* < .001). Hence, in view of pharmacokinetics, TA released from the PEGylated prodrug exhibited more desirable PK profiles. The increased systemic exposure, decreased clearance and prolonged resistant time, which may further guarantee the enhanced level and long circulation of TA *in vivo*, then promoted its anti-tumor efficacy.

### Safety assay of mPEG2K-SA-TA

#### Hemolytic test

**Figure 5.**
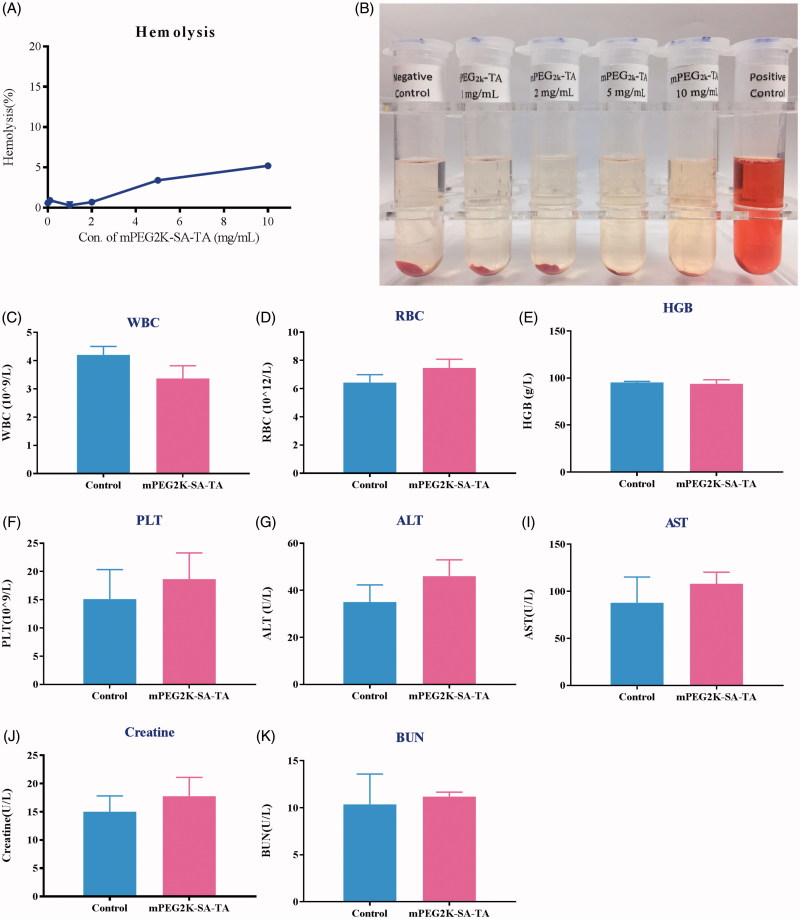
Hemolytic test of drug-free mPEG2K-SA-TA solution (1, 2, 5 and 10 mg/mL). The photo was taken after 3 h reaction (A). Negative control was treated with physiological saline, positive control was treated with distilled water (B); Acute toxicity evaluations in BALB/c mice treated with PEG2K-SA-TA solution (C–K), including blood test (WBC, HGB, RBC and PLT) and serum biochemical index analysis (ALT, AST, CREA, and BUN).

As a synthesized polymer, it was necessary to investigate the potential effect of its surface activity on the cellular membrane integrity, further to determine whether mPEG2K-SA-TA was safe for administration. As shown in Figure 5(A,B), there were no significantly hemolysis occurred in negative control (physiological saline) and mPEG2K-SA-TA (1, 2, 5 and 10 mg/mL). Hemoglobin release was restricted within 1% till the amount of polymer reached 5 mg/mL. The negligible hemolytic activity suggested that mPEG2K-SA-TA was suitable for administration.

#### Acute toxicity evaluation

After i.v. administration of mPEG2K-SA-TA at 1000 mg/kg, there was no mortality in the BALB/c mice up to 14 days. Also, there were no observations of abnormal behaviors. The H&E straining also depicted that mPEG2K-SA-TA did not induce any histological damage to the heart, liver, spleen, lungs or kidneys in BALB/c mice. Moreover, no obvious differences were observed in blood test (WBC, HGB, RBC, and PLT) and biochemical indexes analysis (ALT, AST, Crea, BUN) when compared to the control group (Figure 5(C–K)). Therefore, the synthesized polymer, mPEG2K-SA-TA could be probably safe with no significant damage to liver or kidney, which were also supported by the H&E staining of representative tissues (Figure S3).

#### Preparation and characterization of mPEG2K-SA-TA micelle

**Figure 6.**
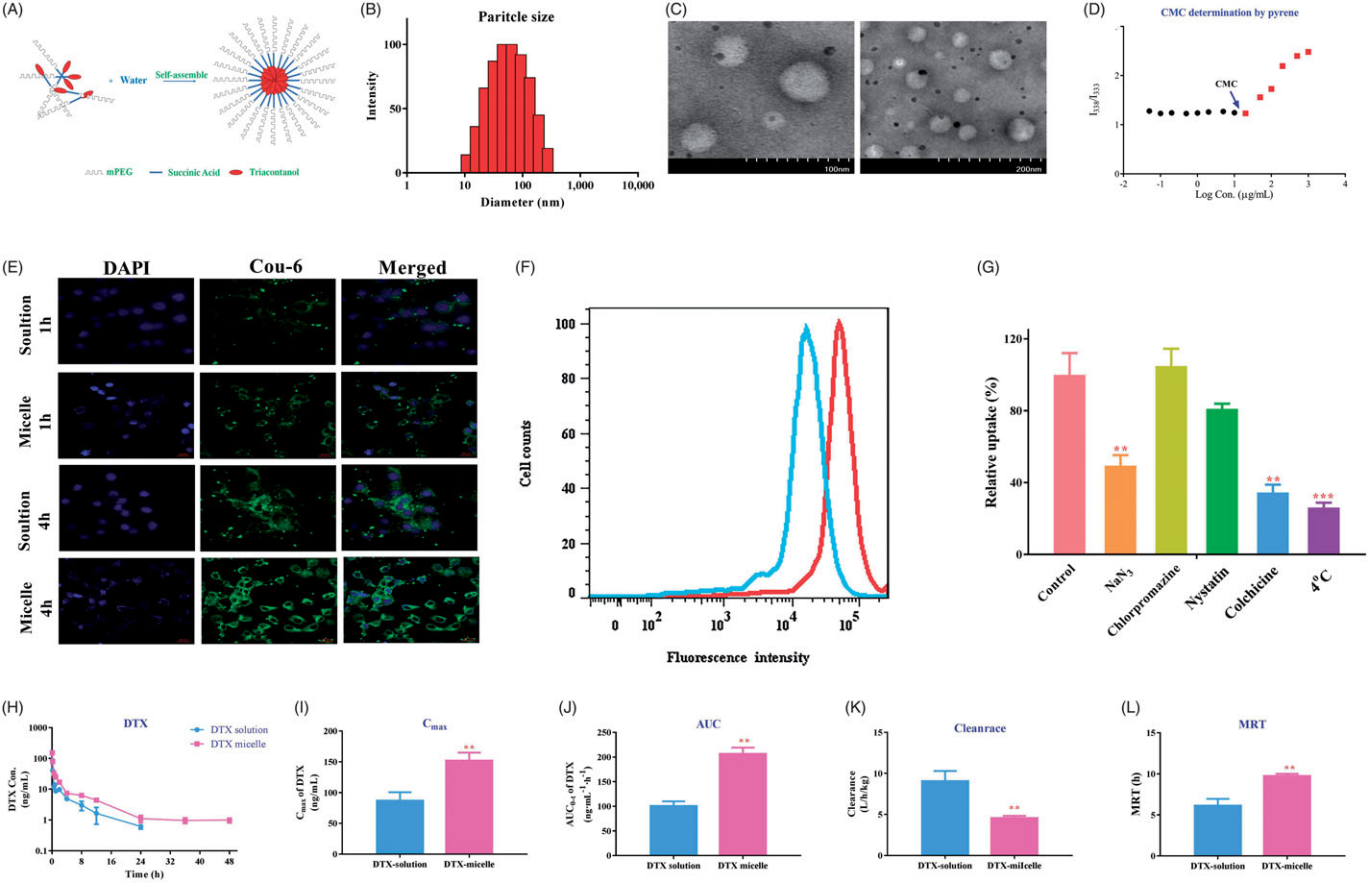
Characterization of PEGylated TA polymer micelles (A); Particle size distribution (B); TEM images (the scale bar at 100 and 200 nm) (C); CMC determination using pyrene as a fluorescence probe (D);. CLSM images of LoVo cells after 1 h or 4 h of incubation with mPEG2K-SA-TA polymer-loaded Coumarin-6 or free Coumarin-6 solution (scale bar at 20 μm) (E); Fluorescence intensity of Coumarin-6 in LoVo cells treated with Coumarin-6 solution of mPEG2K-SA-TA micelle loading Coumarin-6 obtained by flow cytometry (F); Cellular relative uptake of mPEG2K-SA-TA polymer in LoVo cells after 2 h-incubation at 4 or 37 °C with the presence of various inhibitors (including NaN_3_, chlorpromazine, nystatin and colchicine) or not (control group) (G); Plasma concentration–time profiles of DTX in rats following intravenous administration of DTX solution or DTX micelle (encapsulated by mPEG2K-SA-TA) at 1 mg/kg (H); Pharmacokinetic parameters after i.v. administration of the DTX loaded by mPEG2K-SA-TA micelles and DTX solution at a dose of 1 mg/kg (mean ± SD, *n* = 4) (I–L).

Besides its anti-malignant effects, the synthesized mPEG2K-SA-TA also exhibited amphipathic characteristics, in which TA functioned as the hydrophobic end and PEG segment as the hydrophilic end, as depicted in Figure 6(A). After attaching PEG, the synthesized mPEG2K-SA-TA conjugate characterized good water solubility and could be easily self-assembled to micelle in solution, as schematic diagram shown in Figure 6(A), showing the classical core–shell structure. The average particle sizes of blank mPEG-2k-SA-TA micelles were around 56.5 nm with narrow particle size distribution (Polydispersity, 0.237), and exhibited a positive surface charge state (13.08 mv). Its particle size distribution is shown in Figure 6(B). Its morphology was further confirmed by TEM, as shown in Figure 6(C), homogeneous spherical particles were observed, and the particle size was in agreement with that detected using the DLS. Usually, the smaller size (<100 nm) could avoid rapid renal excretion and facilitate cellular uptake via endocytic internalization pathways, but also guarantee the raised accumulation in tumor by enhanced permeability and retention effect (EPR). After a period of storage at 4 °C, the particle size, PDI and zeta potential remained relatively stable, further supported the high stability of the mPEG2k-SA-TA micelles.

Since the spherical morphology and small particle sizes, the point of abrupt change (named as CMC) was measured in order, using pyrene as a fluorescence probe. At lower concentrations of the specified polymer (from 0.05 to 20 μg/ml), the amount of pyrene dissolved in aqueous solution remained stable, as the horizontal line in Figure 6(D). Once the concentration reached the CMC, the hydrophobic part (TA) of polymer aggregated to form the colloidal assemblies, then to generate the hydrophobic core, while the PEG chain was retained in the aqueous solution to form hydrophilic shell, eventually self-assembled to stable core–shell structure. During the process of micelle formation, the pyrene was gradually secured in the hydrophobic core, therefore, the fluorescence intensity of pyrene was substantially increased when the concentration of polymer surpassed the CMC. The cross point between the two lines was designated as CMC, estimated as 19.1 μg/mL for mPEG2k-SA-TA, as shown in Figure 6(D). The low CMC probably provided good stability for drug delivery, even suffered from dilution by circulatory blood *in vivo*. The easier preparation, lower CMC indicated mPEG2K-SA-TA as a good material for drug delivery.

#### Cellular uptake of mPEG2K-SA-TA micelles

In the pH 7.4 PBS solution, the hydrolysis of PEGylated prodrug was minor, there were still 81.4% as intact prodrug in solution up to 48 h, indicating that the synthesized prodrug remained stable in neutral solution. Similar situation was also observed in pH 5.0 solution, as shown in Figure S4. Therefore, mPEG2K-SA-TA remained relatively stable in the incubation mixture, and probably entered the intracellular fluid as intact form. The cellular uptake of polymer prodrug micelle was investigated by loading coumarin-6 in mPEG2K-SA-TA micelle in LoVo cells incubated for 1 and 4 h. Under CLSM observing, the nuclei were stained blue with DAPI, while coumarin-6 emitted green fluorescence indicated the cellular uptake and distribution. Incubating for 1 and 4 h, the green fluorescence was rapidly observed in the cytoplasma of LoVo cells, and the intracellular fluorescence intensity was increased along with the incubation time, also higher fluorescence intensity was observed compared to free coumarin-6 solution shown in Figure 6(E), which further indicated enhanced cellular uptake of coumarin-6 loading by mPEG2K-SA-TA micelles. In accordance with the CLSM result, the intracellular fluorescence intensity of micelle group was higher than that of solution group, detected by flow cytometry as shown in Figure 6(F). The intracellular uptake of micelle indicated that the synthesized PEGylated TA could efficiently internalize into tumor cells, mainly as intact form.

#### Endocytosis pathway investigation of mPEG2K-SA-TA micelle

Traditionally, nanoparticles predominately enter the cells via endocytosis pathway (Sahay et al., 2010; Yameen et al., 2014). mPEG2K-SA-TA was able to self-assemble to micelle in solution, with smaller particle diameters. Therefore, the possible mechanism of internalization of polymer micelles was necessary to be investigated via co-incubating the micelle with various endocytic inhibitors. NaN_3_ is able to inhibit the ATP-dependent endocytosis pathway (Schmid & Carter, 1990), chlorpromazine restrains clathrin-mediated endocytosis (Rejman et al., 2005), colchicine suppresses the macropinocytosis pathway (Kruth et al., 2005), and nystatin impedes the caveolin-dependent endocytosis (Zhang et al., 2018). When co-incubated with NaN_3_, the amount of polymer micelle was significantly decreased 49.5% compared to the cells with no inhibitors treatment, and the low-temperature (4 °C) also reduced the intracellular amount of polymer micelle (only 26.2%, vs. control cell). Both indicated that the endocytosis process of polymer conjugate was energy-dependent. When LoVo cells were incubated with colchicine, the entrance of polymer micelle was significantly blocked, demonstrating the involvement of macropinocytosis in cellular uptake of polymer, as depicted in Figure 6(G). While nystatin and chlorpromazine exhibited no obvious inhibition on the micelle uptake.

#### mPEG2K-SA-TA micelle-load DTX

In consideration that the synthesized polymer TA micelle was able to efficiently carry coumarin-6 to cells, it also encapsulated DTX in micelle to delivery DTX. The particle size of the DTX-loaded micelles was in the range of 88.7 ± 1.5 nm with a narrow particle size distribution (PDI, 0.225 ± 0.007), shown in Figure S5A. The particle size of DTX micelles was approximately within 100 nm, the smaller particle size avoids rapid renal clearance. The morphology of micelles loaded with DTX was consistent with the morphology of the blank micelle which was still a spherical structure (as depicted in Figure S5B). After determination, the entrapment efficiency reached 91.51 ± 8.54%, while the drug loading of DTX was 1.79%.

Afterwards, the pharmacokinetic features of DTX with different formulations (DTX-solution, DTX-micelle) were studied after i.v. administration in rats. The plasma concentration-time profiles were depicted in Figure 6(H) and the pharmacokinetic parameters were compared and summarized in Figure 6(I–L), including AUC_0−t_, *C*_max_, clearance and MRT. By comparison to DTX-solution administration, the DTX-micelle resulted in significantly increased systemic exposure to DTX (206.3 vs. 96.19 ng·mL^−1^·h^−1^, *p* < .01). Furthermore, the clearance in DTX-micelle group was obviously decreased to 4.89 L·h^−1^·kg^−1^ (*p* < .01), while its counterpart treated with DTX-solution was 10.8 L·h^−1^·kg^−1^. These results suggested that the polymer micelle successfully loaded DTX and played a key role in raising systemic exposure and decreasing clearance of DTX, which could guarantee the anti-tumor potency of DTX.

## Conclusions

After grafted with mPEG, PEGylated TA was readily soluble in aqueous solution and promisingly enhanced the pharmacokinetic property of TA, which may contribute to its enhanced therapeutic effects. PEGylated TA micelle exhibit its anti-malignant effects via the inhibition of NF-κB nuclear translocation, suppression on MMPs and angiogenic signaling (summarized in Figure S6). Besides anti-tumor potency, PEGylated TA micelle was also able to encapsulate docetaxel to alleviate its pharmacokinetic process. Therefore, the PEGylated TA owned bifunctionality as anti-tumor agents and solubilizers, hereby, it warrants further investigations.

## Supplementary Material

Supplementary_materials-revised.doc
