# Supplementary material for: Superior anti-neoplastic activities of triacontanol-PEG conjugate: synthesis, characterization and biological evaluations
Source: Drug Deliv. 2018 Jul 19;25(1):1546–59. doi: 10.1080/10717544.2018.1477864 (PMC6060375; doi:10.1080/10717544.2018.1477864)

**Supporting information for western blot analysis**

According to the manufacturer’s instructions, the lysis buffer was added to lyse cells and extract the protein by the commercial nuclear and cytoplasmic protein extraction kit (Beyotime Institute of Biotechnology, Shanghai, China). The extraction process was performed on ice to reduce protein degradation. The harvested supernatant was immediately stored at −80 °C till western blotting analysis. An aliquot was pipetted to determine the protein content by BCA method (commercial kits were purchased from Nanjing Jiancheng Bioengineering Institute, Nanjing, China). As conducted in the *in vitro* cells, the tumor tissues (collected from BALB/c nude mice bearing LoVo cells) were prepared with similar process via commercial nuclear and cytoplasmic protein extraction kit. Afterwards, the extracted proteins (protein loading amount, 75 μg) were separated on 10% SDS-PAGE electrophoresis (electrophoresis using Bio-Rad, Hercules, CA) and transferred to PVDF membranes (0.45 μm pore size, Pall, USA), they were further blocked in 5% nonfat milk for 2 h and incubated with different primary antibodies at 4 °C overnight. PVDF membranes were washed three times with 0.5% TBS-T solution (5 min each time). Afterwards, the HRP-labeled secondary antibody was reacted and washed with 0.5% TBS-T solution three times, each time for 5 min. The membrane was incubated with lumiGLO reagent (Cell Signaling, Beverly, MA) and the bands were visualized with an X-ray (Kodak, New Haven, CT) using analytical software (Bio-Rad Laboratories, Hercules, CA) analysis.

**Supporting information for Real-time qPCR analysis**

The total RNAs of LoVo cells were extracted using Trizol reagent (Servicebio, Wuhan, China). Referring to the user guide, the cDNA was synthesized using the RevertAid First Strand cDNA Synthesis Kit (Thermo Fisher Scientific, MA, USA). The qPCR was performed using FastStart Universal SYBR Green Master (Roche, Basel, Switzerland) and conducted by Real-Time PCR System (Thermo Fisher Scientific, MA, USA). The relative values of mRNA were normalized with the levels of GAPDH mRNA and the relative gene expressions were assayed with the 2−∆∆Ct method. The mRNA levels of both MMP9 and VEGF in LoVo cells were determined. In tumor tissues from nude mice bearing LoVo cells, the mRNA levels of MMP9 and VEGF were analyzed as the identical procedure.

**Supplementary Table 1 The primer sequences for Q-PCR analysis of MMP9 and VEGF in tumors and LoVo cells**

| Primer | Primer sequence |
| --- | --- |
| Forward GAPDH | ACTTTGGTATCGTGGAAGGACTCAT |
| Reverse GAPDH | GTTTTTCTAGACGGCAGGTCAGG |
| Forward β-actin | CACCCAGCACAATGAAGATCAAGAT |
| Reverse β-actin | CCAGTTTTTAAATCCTGAGTCAAGC |
| Forward-VEGF | GGAGGGCAGAATCATCACGA |
| Reverse VEGF | GCTCATCTCTCCTATGTGCTGG |
| Forward MMP9 | CGAACTTTGACAGCGACAAGA |
| Reverse MMP9 | TTCAGGGCGAGGACCATAGAG |

**Supplementary Figure 1** Body weight changes of LoVo tumor-bearing nude mice after treatment with saline or different drugs for 21 successive days

**
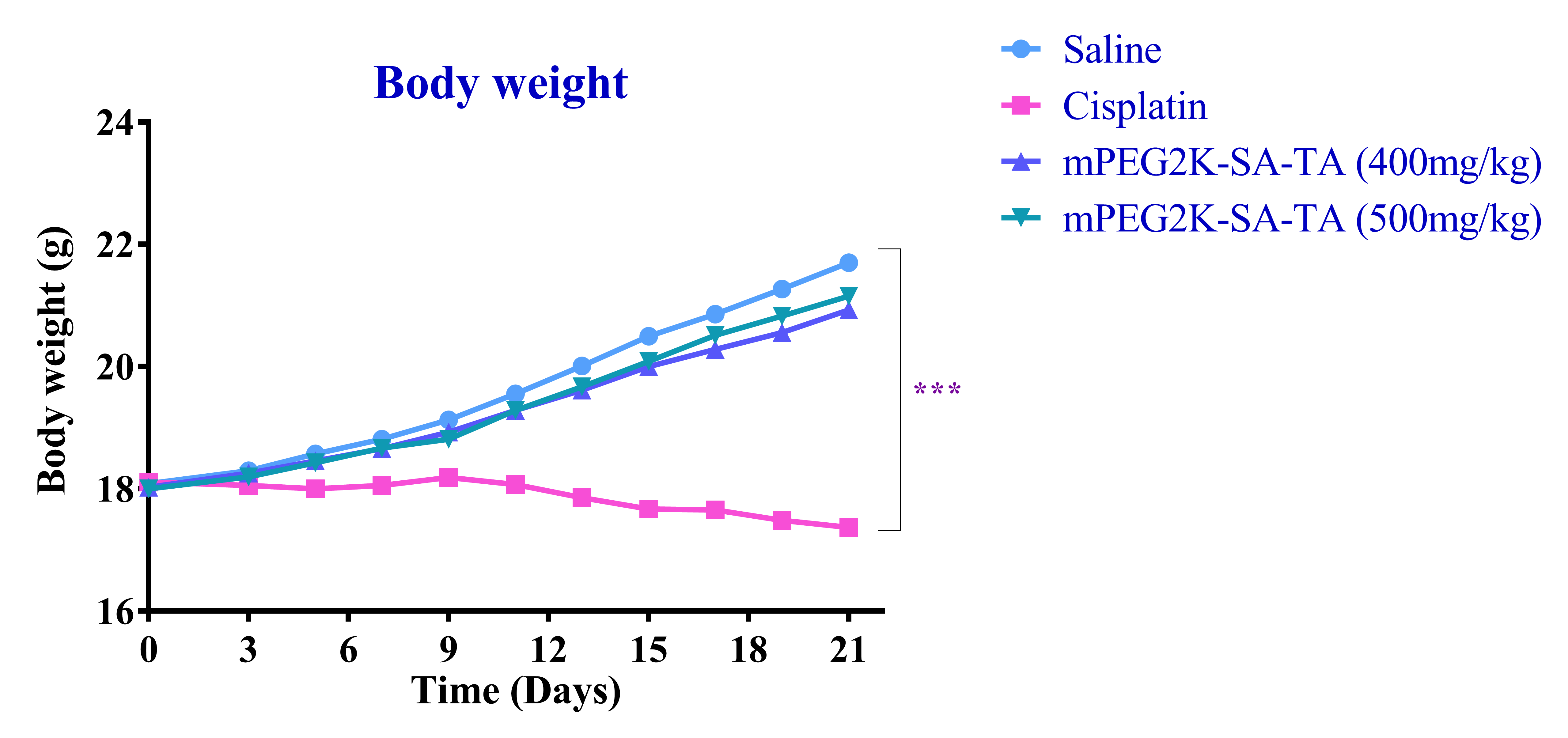
**

**Supplementary Figure 2** Representative MMP9 and VEGF immunohistochemical images of LoVo tumor tissues from nude mice treated by mPEG2K-SA-TA for 21 successive days

**
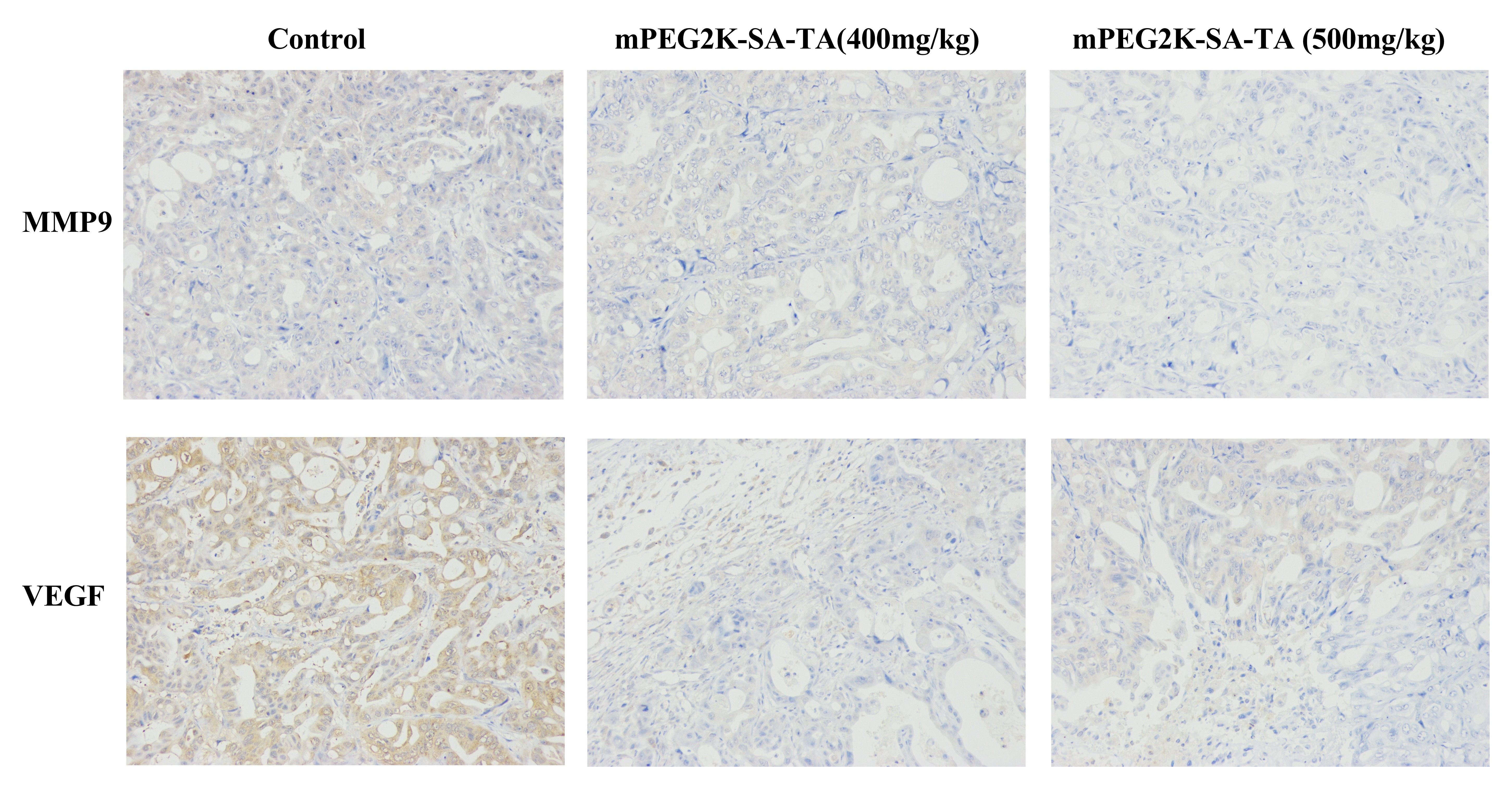
**

**Supplementary Figure 3** H&E staining images of the vital organs of mice after treatment with saline, mPEG2K-SA-TA (1000 mg/kg)


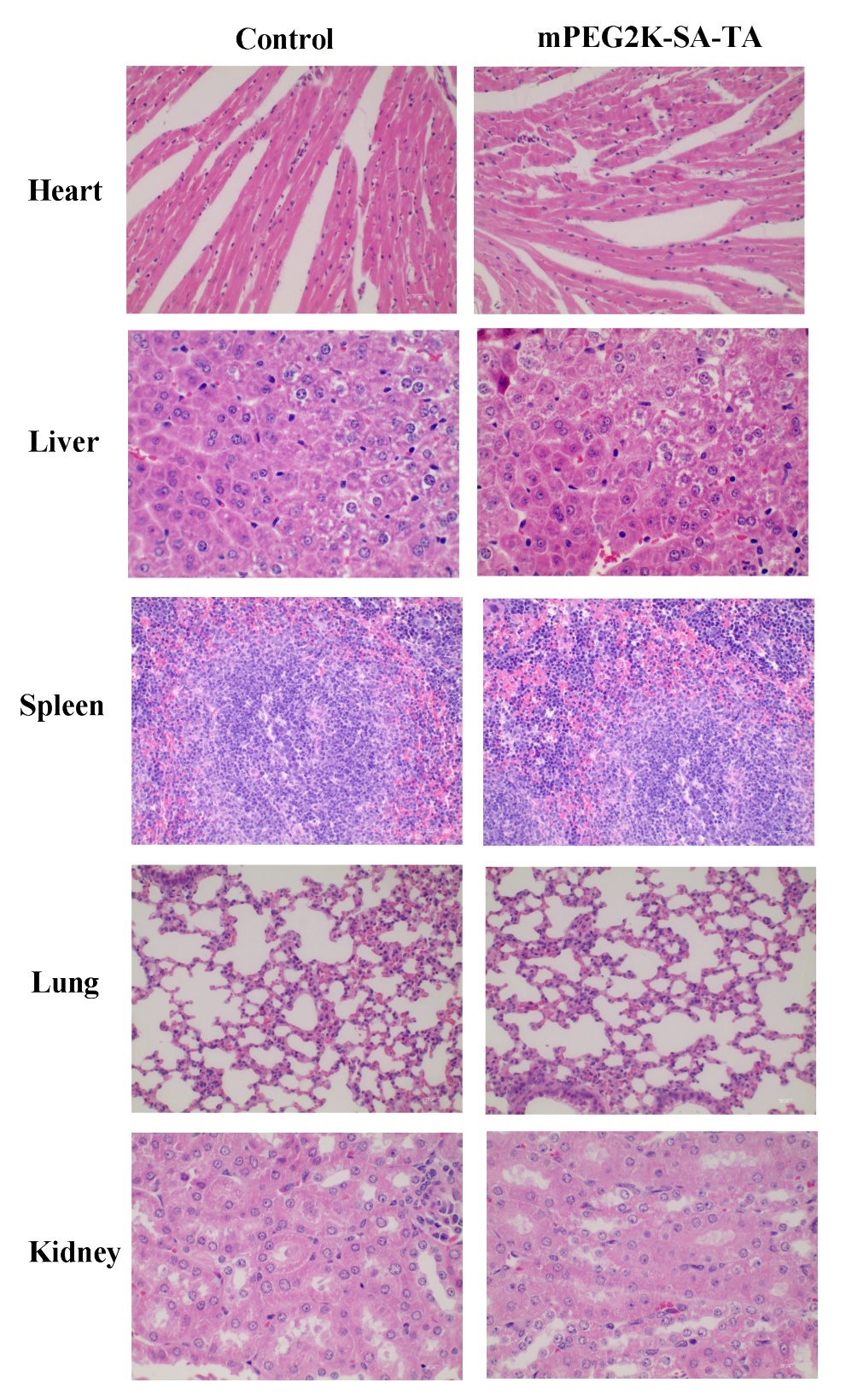


**Supplementary Figure 4** *In vitro* stability profiles of PEGylated TA micelle (calculated by released TA) in PBS solution containing 0.5% Tween 80 at pH 7.4 and 5.0


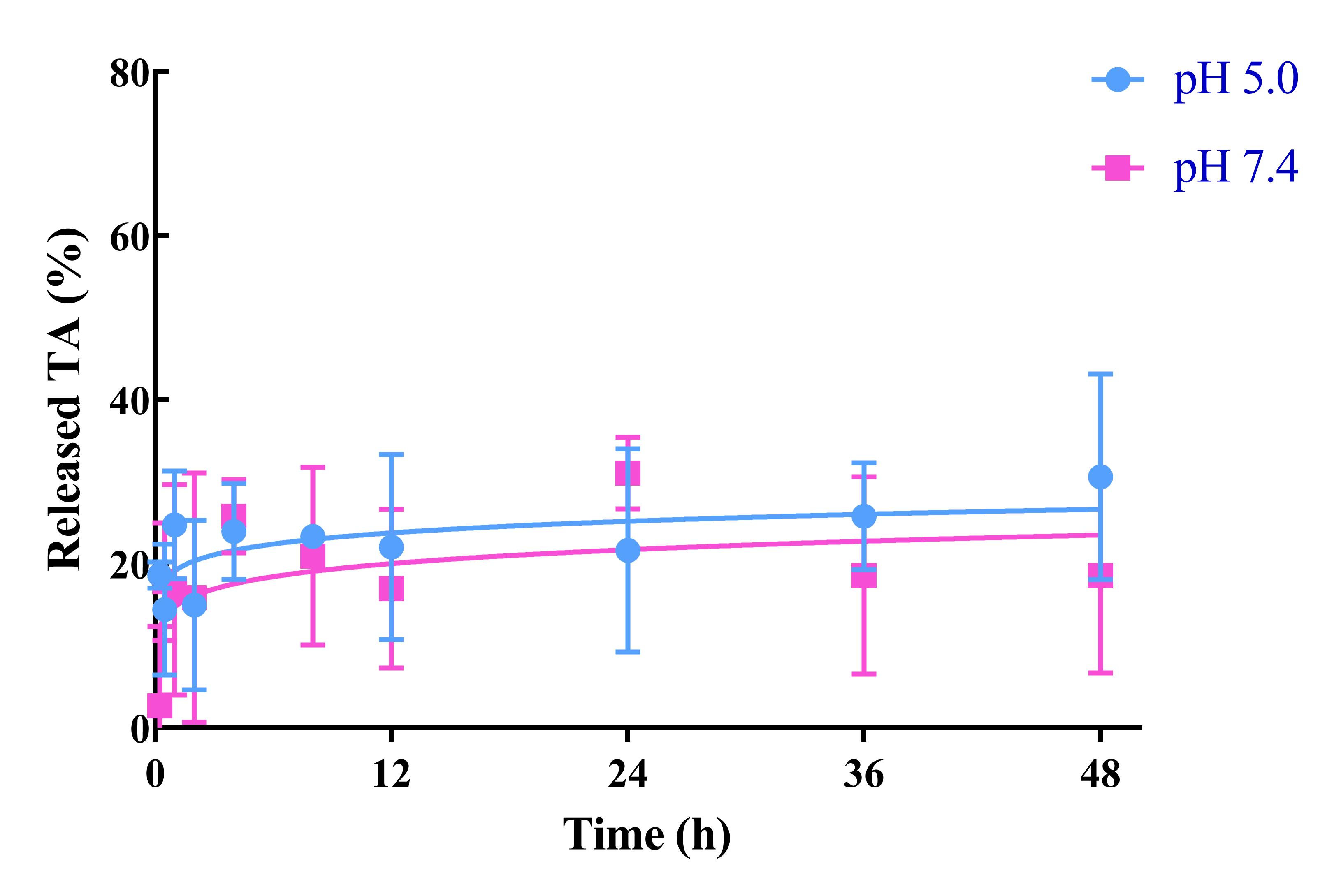


**Supplementary Figure 5** Particle size distribution **(A)** and TEM images **(B)** of mPEG2K-SA-TA micelle loading DTX

**
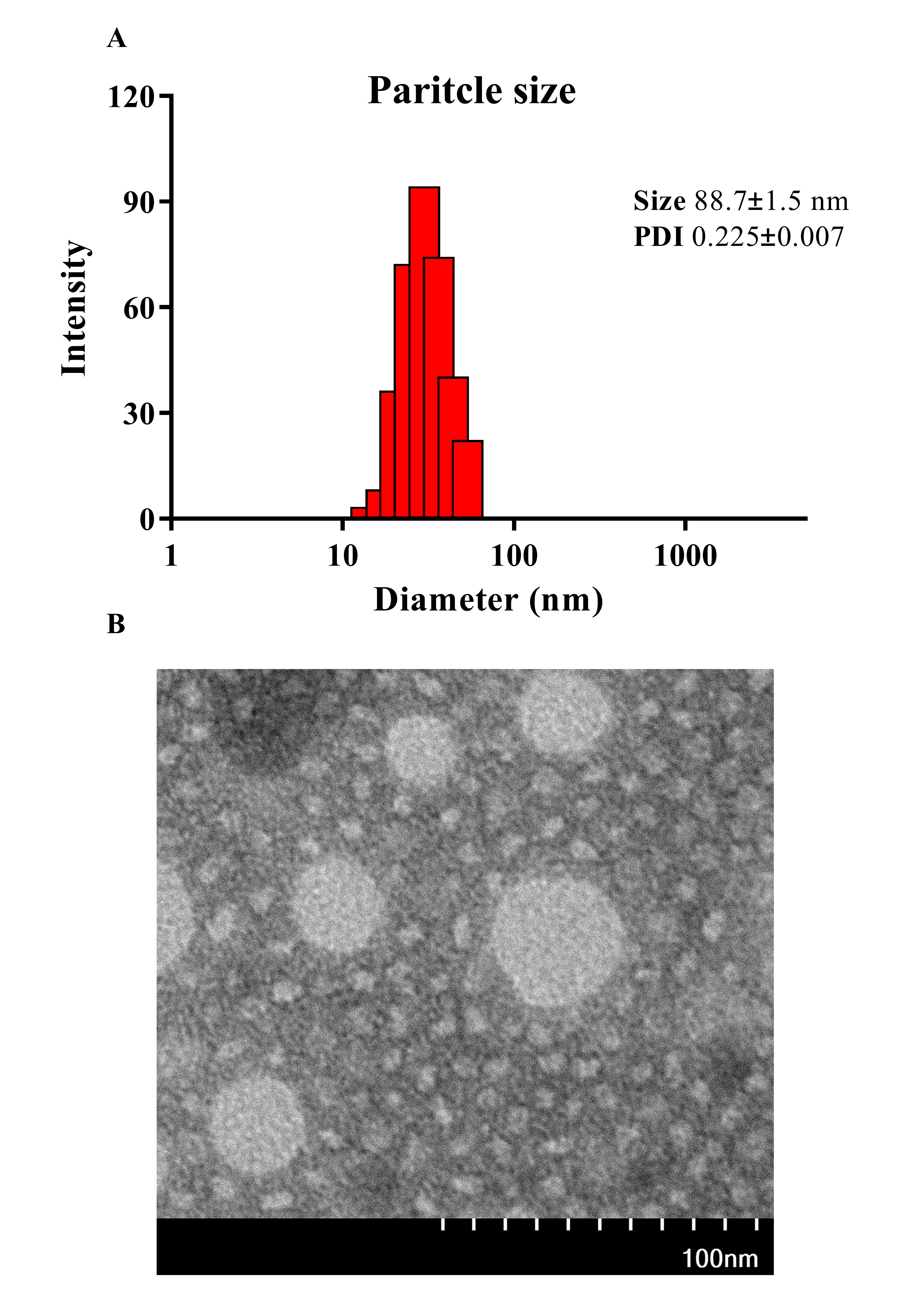
**

**Supplementary Figure 6** Graphic abstract of present study—The self-assembled PEGylated TA micelle was predominately internalized into cells via energy-dependent macropinocytosis, in which the PEGylated TA was degraded to TA to exhibit the anti-tumor potency via NF-κB translocation inhibition


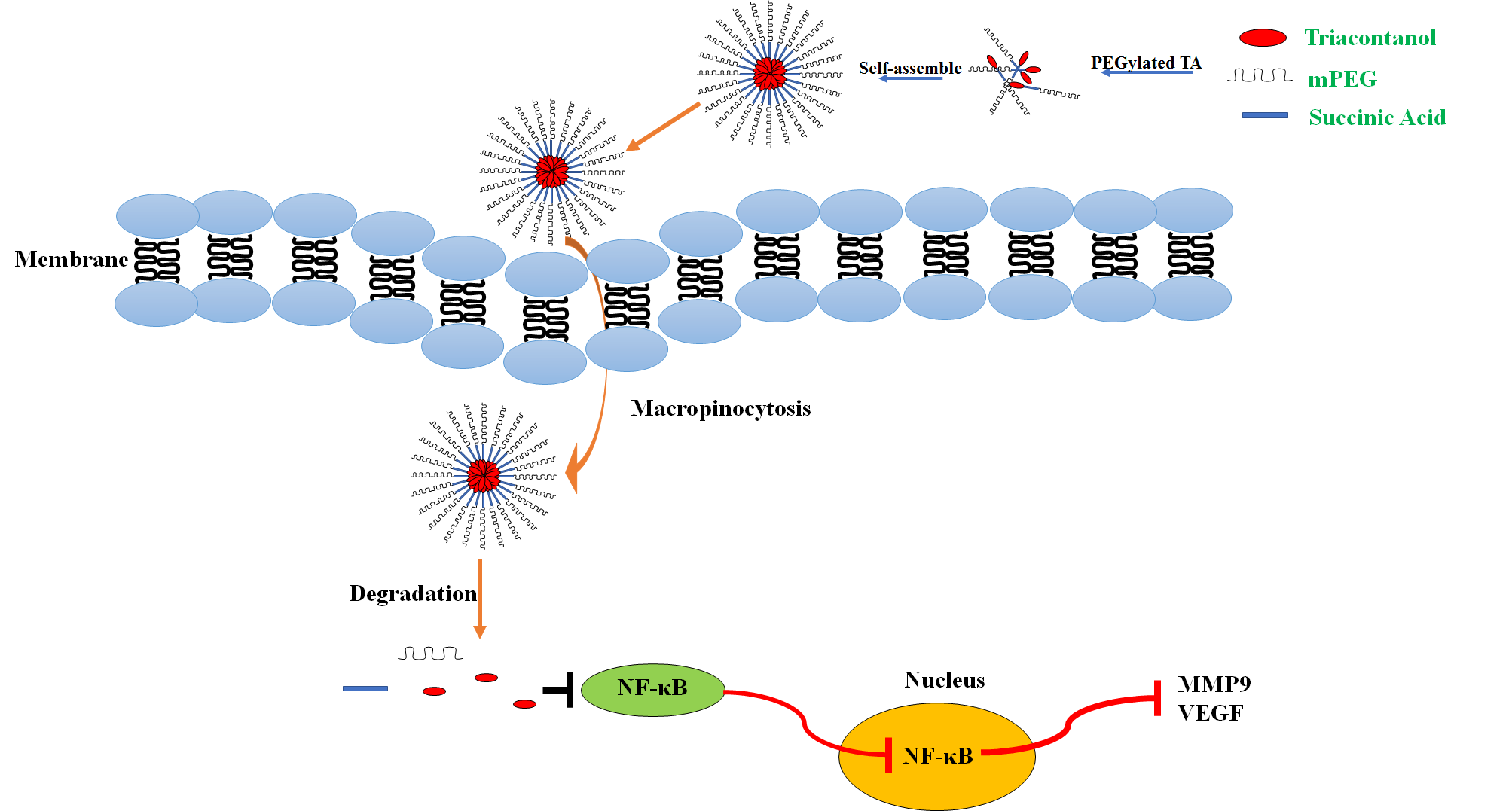

Supplement: Supplementary_materials-revised.doc [file IDRD_A_1477864_SM6351.doc]
